# Physiology-Based Diagnosis and Management of Bronchopulmonary Dysplasia Associated Pulmonary Hypertension (BPD-PH)

**DOI:** 10.3390/children13020272

**Published:** 2026-02-15

**Authors:** Yogen Singh, Sfurti Nath, Sheen Gahlaut, Belinda Chan

**Affiliations:** 1 Division of Neonatology, Department of Pediatrics, University of California Davis Health, 2516 Stockton Blvd, Sacramento, CA 95817, USA; belchan@health.ucdavis.edu; 2 Department of Pediatrics (Neonatology), Women and Infants Hospital, The Warren Alpert Medical School of Brown University, Providence, RI 02905, USA; snath@kentri.org; 3 Lincoln College, Oxford University, Oxford OX1 3DR, UK; sheen.gahlaut@lincoln.ox.ac.uk

**Keywords:** bronchopulmonary dysplasia (BPD), pulmonary hypertension (PH), Bronchopulmonary associated pulmonary hypertension (BPD-PH), neonatal echocardiography, phenotypes, pathophysiology, screening algorithm, vascular remodeling

## Abstract

Bronchopulmonary dysplasia (BPD) remains a major long-term morbidity among preterm infants. As lung-protective strategies advance and survival of extremely premature neonates improves, BPD has evolved from a ventilator-induced inflammatory and fibrotic process to a disease marked by arrested pulmonary vascular and alveolar development—pulmonary vascular disease. Within this evolving phenotype, pulmonary hypertension (PH) has emerged as a critical yet underrecognized complication. BPD-associated pulmonary hypertension (BPD-PH) is increasingly linked to higher mortality and worse clinical outcomes, but its pathophysiology, screening strategies to detect early changes, and optimal management remain incompletely understood. This review delineates the pathophysiology of BPD-PH, linking impaired pulmonary vascular development with subsequent maladaptation influenced by genetic, prenatal, and postnatal factors. The phenotypic and hemodynamic spectrum of BPD-PH is further subclassified using echocardiographic markers to support a physiology-based approach to diagnosis and management. We also propose a pragmatic algorithm for screening, evaluation, and longitudinal follow-up. Collectively, this review highlights the need for physiology-driven strategies and clinical studies to improve outcomes in these neonates.

## 1. Introduction

Bronchopulmonary dysplasia (BPD) remains the most common complication of prematurity and is associated with lifelong respiratory, cardiovascular, and neurodevelopmental impairments that compromise survival and quality of life [1,2]. Severe BPD is particularly debilitating and may shorten life expectancy. Contemporary cohorts estimate that 40–68% of infants born at ≤28 weeks’ gestation develop BPD, with prevalence rising as survival improves among extremely preterm neonates—especially those born at 22–24 weeks, who carry the greatest risk of severe disease [3,4,5,6].

The pathophysiology of BPD has evolved in parallel with its epidemiology. The “old” phenotype was characterized by ventilator-induced inflammation and fibrosis in relatively mature preterm lungs [7]. However, advances in ventilator strategies and neonatal care have enabled neonates to survive at earlier gestational ages, shifting the disease toward a “new” phenotype. This “new” BPD is defined by arrested alveolar and pulmonary vascular development in extremely immature lungs exposed to cumulative prenatal and postnatal injury [8,9,10,11,12,13].

Within this evolving phenotype, pulmonary vascular disease has emerged as a key driver of outcomes, giving rise to BPD-associated pulmonary hypertension (BPD-PH). BPD-PH is now understood as a complex lung–vascular–cardiac disorder with important hemodynamic consequences [1,2]. The prevalence of BPD-PH rises with disease severity, affecting ~5% of infants with mild BPD, 18% with moderate BPD, and over 40% with severe disease [14]. BPD-PH confers a several-fold increase in mortality with strong associations to adverse neurodevelopmental outcomes compared to infants with BPD alone [15]. Nearly half of extremely preterm neonates with BPD-PH die within two years of diagnosis [16,17].

Beyond mortality, BPD-PH imposes substantial morbidity, including prolonged respiratory support, extended hospitalization, tracheostomy, increased caregiver burden, decreased pulmonary function, decreased exercise tolerance, and greater healthcare utilization [4,18,19]. Although pulmonary hypertension resolves in about 47% by 1 year and in 79–94% by 2–2.25 years [17], affected neonates remain vulnerable to hemodynamic instability during a critical window of neurodevelopment. These risks underscore the urgency of early recognition and physiologic risk stratification to target treatment.

BPD-PH is a heterogeneous disorder characterized by diverse phenotypes and hemodynamic manifestations. Subclassification using echocardiographic markers may enable physiology-based, early targeted interventions. However, consensus on phenotypic definitions and severity classification in neonates is still lacking [20,21,22,23]. Although current guidance from the American Heart Association/American Thoracic Society (AHA/ATS), the European Pediatric Pulmonary Vascular Disease network (EPPVAD), the World Symposium on Pulmonary Hypertension (WSPH), and the European Respiratory Society (ERS) provides important frameworks for PH, these recommendations are largely derived from adult and pediatric populations and may not fully reflect the unique pathophysiology of neonates with BPD-PH.

This review proposes a novel, phenotype-driven approach to BPD-PH that integrates diagnostic evaluation with therapeutic algorithms to support more individualized care. Such a strategy has the potential to improve both short- and long-term outcomes in this highly vulnerable population.

## 2. Pathophysiology of BPD and PH in BPD

**Figure 1:**
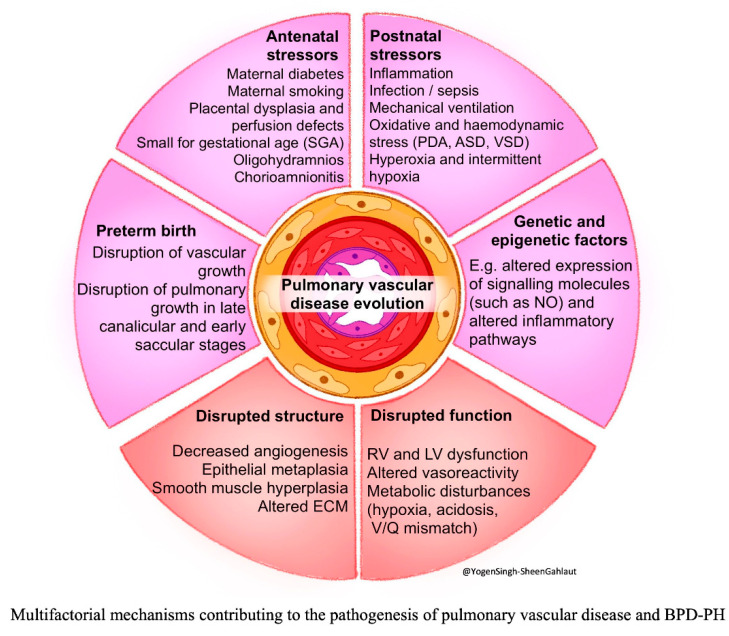
Multifactorial mechanisms contributing to the pathogenesis of pulmonary vascular disease and bronchopulmonary dysplasia associated pulmonary hypertension (BPD-PH). Abbreviations: ASD, atrial septal defect; ECM, extracellular matrix; LV, left ventricle; NO, nitric oxide; PDA, PDA patent ductus arteriosus; RV, right ventricle; SGA, small for gestational age; VSD, ventricular septal defect; V/Q, ventilation/perfusion. (Figure courtesy of @Yogen Singh-Sheen Gahlaut).

The pathophysiology of BPD and BPD-PH is multifactorial and summarized in Figure 1. Contributing factors can be broadly categorized as: (a) antenatal stressors, (b) preterm birth, (c) postnatal stressors, (d) disrupted lung and vascular structure, (e) molecular and signaling pathways, and (f) hemodynamic stressors and disrupted function.

### 2.1. Antenatal Stressors

Prenatal exposures such as maternal diseases, fetal growth restriction, placental insufficiency, oligohydramnios, and chorioamnionitis occur during a pivotal stage of fetal lung morphogenesis. These disruptions interfere with coordinated secondary septation and angiogenesis required to form normal alveolar–capillary interfaces, which increase the risk of BPD and BPD-PH [24,25,26].

### 2.2. Preterm Birth

Extremely preterm neonates are delivered during the canalicular or early saccular stages of lung development, prior to the onset of alveolarization, leaving the immature lungs particularly susceptible to postnatal injury and impaired developmental progression [26].

### 2.3. Postnatal Stressors

Postnatally, exposure to mechanical ventilation, inflammation, infection, oxidative stress from hyperoxia or hypoxia, and hemodynamic perturbations further impair normal lung and pulmonary vascular development [24,25,26,27].

### 2.4. Disrupted Lung and Vascular Structure

During normal lung development, the double-layered alveolar capillary network remodels into a near single-layered structure with coordinated septation as alveoli mature. In BPD, the persistence of this immature architecture with disrupted septation results in fewer, larger alveoli with a reduced gas-exchange surface area [25,28,29,30]. Impaired septation also interferes with angiogenesis, leading to pulmonary vascular rarefaction and a diminished vascular bed. Subsequent compensatory structural remodeling—including medial hypertrophy and elastic lamina fragmentation—further increases vascular tone, pulmonary vascular resistance (PVR), and pulmonary arterial pressure (PAP). Together with parenchymal and airway injuries, these abnormalities promote hypoxic vasoconstriction, impaired pulmonary blood flow (PBF), and progressive vascular remodeling, ultimately predisposing infants to BPD-PH [26,28].

### 2.5. Molecular and Signaling Pathways

Pulmonary vascular development is tightly regulated by vascular endothelial growth factor (VEGF) and endothelial nitric oxide synthase (eNOS) signaling, which maintain endothelial integrity, promote angiogenesis, and support alveolar–capillary coupling. Oxidative stress and inflammation disrupt these pathways by suppressing VEGF activity and reducing nitric oxide bioavailability, leading to impaired capillary growth, and the uncoupling of alveolar and vascular development. Animal studies further underscore the importance of endothelial-derived “angiocrine” signaling in driving alveolar epithelial growth and septation, highlighting these pathways as promising therapeutic targets [27,28,29,30,31,32].

Multiple additional molecular regulators contribute to BPD-PH pathogenesis, including insulin-like growth factor-1, angiopoietin–TIE-2 signaling, sphingosine-1-phosphate receptor pathways, and extracellular matrix and inflammatory mediators such as IL-6, TNF-α, and TGF-β [27,31,32,33,34,35,36]. Experimental modulation of these networks alters endothelial progenitor cell function and angiogenesis, reinforcing their central role in disease development.

### 2.6. Hemodynamic Stress and Disrupted Function

Physiological or intracardiac shunts can increase hemodynamic stress and contribute to BPD-PH. Extremely preterm neonates are at high risk for persistent patent ductus arteriosus (PDA), resulting in pulmonary overcirculation [37]. Both the duration and severity of PDA shunt exposure have been associated with the development of BPD-PH at discharge [10]. Other cardiac shunts—including interatrial shunt via patent foramen ovale (PFO) or atrial septal defect (ASD) and ventricular septal defect (VSD)—have also been linked to an increased risk of BPD-PH compared with matched controls [27]. Observational studies suggest that early closure of ASD may attenuate PH severity in select infants [10].

Cardiopulmonary interactions can exacerbate the severity of BPD-PH. As PAP and PVR increase, the right ventricle (RV) faces chronic pressure overload, leading to hypertrophy, dilation, and progressive systolic and diastolic dysfunction. Ventricular interdependence further amplifies this process, as RV dilation distorts left ventricular (LV) geometry, impairing filling and reducing cardiac output, ultimately resulting in global cardiac dysfunction. Additionally, pulmonary overcirculation from PDA and intracardiac shunts may impose further volume stress on the LV. Echocardiography is therefore indispensable for assessing ventricular performance and hemodynamic compromise.

## 3. Phenotypes of BPD-PH

BPD-PH is a multifactorial disorder with heterogeneous clinical presentations and diverse underlying mechanisms. Emerging frameworks conceptualize BPD-PH along a spectrum ranging from a vasoreactive phenotype—characterized by elevated vascular tone and potentially reversible dysfunction responsive to vasodilator therapy—to a structural phenotype marked by pulmonary vascular hypoplasia and fixed remodeling with limited treatment responsiveness. Importantly, indiscriminate use of vasodilators in certain phenotypes, such as those with PDA and significant pulmonary overcirculation, may worsen clinical status. These considerations underscore the importance of phenotype delineation; however, standardized definitions are lacking.

Major PH societies (AHA/ATS, WSPH) first established PH classification for adults based on underlying mechanisms, and later EPPVAD refined it for pediatric population based on underlying mechanisms [19,20,21,22,23]. The seventh WSPH classification from the pediatric task force is as follows [23]:

Group 1. PAH (pre-capillary)

Group 2. PH with left heart disease (post-capillary)

Group 3. PH with lung disease and/or hypoxia (combined pre- and post-capillary)

Group 4. PH with pulmonary artery obstructions

Group 5. PH with unclear or multifactorial mechanisms

Although existing frameworks broadly categorize BPD-PH within Group 3 PH, this approach risks oversimplifying the complex cardiopulmonary physiology of preterm neonates with BPD [38]. A more nuanced classification may better inform clinical decision-making and therapeutic strategies based upon distinct physiology. Recognizing this heterogeneity, we propose a physiology-driven subclassification to support early targeted specific or definitive management, distinguishing the following [38]:

**Type** **1.** Pre-capillary BPD-PH, due to increased pulmonary vascular resistance (PVR);
**Type** **2.** Flow-dependent BPD-PH, due to increased pulmonary blood flow (PBF);
**Type** **3.** Post-capillary BPD-PH, due to increased pulmonary capillary wedge pressure (PCWP):
  - **3a** from left ventricular (LV) dysfunction/mitral regurgitation abnormality;
  - **3b** from pulmonary vein stenosis.

**Figure 2:**
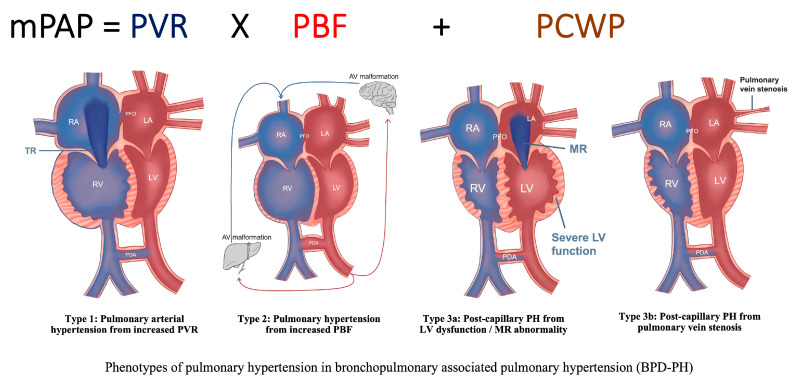
Phenotypes of bronchopulmonary dysplasia associated pulmonary hypertension (BPD-PH). Type 1—pulmonary arterial hypertension from increased pulmonary vascular resistance (PVR), usually from primary lung and pulmonary vascular dysgenesis; Type 2—flow-dependent PH which later can lead to remodeling of pulmonary vasculature and increased PVR; Type 3 (post-capillary type): 3a—post-capillary PH secondary to severe left ventricular (LV) dysfunction leading to increased left atrial (LA) pressure and increased pulmonary capillary wedge pressure (PCWP), and 3b—post-capillary PH secondary to pulmonary vein stenosis (PVS), which is acquired in infants with BPD-PH. Abbreviation: BPD-PH, bronchopulmonary dysplasia associated pulmonary hypertension (BPD-PH); LA, left atrium; LV, left ventricle; mPAP, mean pulmonary artery pressure; MR, mitral regurgitation; PBF, pulmonary blood flow; PCWP, pulmonary capillary wedge pressure; PDA, patent ductus arteriosus; PFO, patent foramen ovale; PVR, pulmonary vascular resistance; RA, right atrium; RV, right ventricle. (Figure courtesy of @Yogen Singh-Sheen Gahlaut).

Central to this framework is the understanding that mean PAP (mPAP), which is determined by PVR, PBF, and PCWP (Figure 2). Thus, elevated PAP may result from increased resistance, excessive PBF, elevated left-sided filling pressures, or a combination of these factors. Anchoring phenotypic classification in these hemodynamic principles facilitates more precise diagnostic evaluation and targeted management.

### 3.1. Type 1 Pre-Capillary BPD-PH, Due to Increased PVR

This is the dominant phenotype in preterm neonates with BPD and occurs primarily due to increased PVR at the arteriolar level from pulmonary vascular maldevelopment and maladaptation (Figure 2). This is characterized by low lung compliance, parenchymal heterogeneity, chronic hypoxemia, elevated vascular tone, and disrupted microvasculature growth. Capillary rarefaction and distal muscularization are noted. Echocardiography shows RV hypertrophy and/or dilatation, tricuspid regurgitation (TR), interventricular septum (IVS) flattening especially during systole, eccentricity index (EI) ≥ 1.3, shortened pulmonary artery acceleration time (PAAT), reduced tricuspid annular plane systolic excursion (TAPSE), and often bidirectional/right to left cardiac shunts (e.g., PDA, PFO, ASD, or VSD). Treatment with oxygenation, optimal lung recruitment and ventilation, and growth support are essential, as are specific pulmonary vasodilator therapies [21,23,39,40].

### 3.2. Type 2 Flow-Dependent BPD-PH, Due to Increased PBF

Some infants with BPD may have persistent PDA, interatrial shunt (via persistent PFO or ASD), VSD, or aorto-pulmonary (AP) collaterals, and any other left-to-right cardiac shunt leading to increased PBF and with increased pulmonary to systemic blood flow ratio (Qp:Qs). Systemic arterial collateral vessels have been reportedly linked to BPD-PH that were identified during cardiac catheterization. Most are small and clinically insignificant, but if the vessels are enlarged, they can cause considerable shunting of blood flow into the lungs, leading to pulmonary edema and the need for higher respiratory support and increased oxygen requirements. In rare cases, embolization of large collateral vessels has improved respiratory status, but it is unclear whether these vessels contribute to the pathophysiology of BPD-PH or are a consequence of disease severity [19].

Persistent pulmonary overcirculation can lead to pulmonary vascular diseases with remodeling of the arterial bed and increased PVR. The echocardiography findings in Type 2 show signs of PH like Type 1. PFO, ASD, and VSD can result in RA and RV volume overload, leading to chamber dilation and septal flattening predominantly during diastole. In contrast, a PDA produces a post-RV left-to-right shunt, causing LA and LV dilation. Pulmonary overcirculation increases pulmonary venous return to the LA, manifested by elevated systolic and diastolic laminar Doppler velocities within the pulmonary veins, often exceeding 50 cm/s in neonates. On lung ultrasonography, there will be signs of increased pulmonary edema (increased B-lines) while chest X-rays show non-homogeneous signs of chronic lung disease/BPD and pulmonary edema. In the short term, diuretics will help reduce pulmonary edema and facilitate intervention to eliminate the cardiac shunt. Unlike Type 1 BPD-PH, pulmonary vasodilators may increase pulmonary blood flow and hence worsen PH.

### 3.3. Type 3—Post-Capillary BPD-PH, Due to Increased PCWP

Type 3 BPD-PH is characterized by elevated pressure distal to the pulmonary vascular bed, reflected by increased pulmonary capillary wedge pressure (PCWP) and pulmonary venous congestion.

Type 3a is less common in neonates with BPD-PH, but it is often seen in neonates with congenital diaphragmatic hernia (CDH) or left heart disease (such as hypoplastic left heart syndrome, mitral stenosis or regurgitation, or left ventricular outflow tract or arch obstruction). The neonate with LV systolic or diastolic dysfunction or mitral valve regurgitation/stenosis will have increased LA pressure and pulmonary venous engorgement as evidenced by increased PCWP. Increased LA pressure may cause secondary increases in left-to-right shunt across the interatrial septum, which may serve as a pop-off mechanism and decrease LA pressure. However, it may also exacerbate pulmonary overcirculation. Management focuses on improving LV function and reducing afterload. Avoid pulmonary vasodilators that may worsen pulmonary edema [20,39].

Type 3b arises from pulmonary vein stenosis (PVS), a distinctive and often acquired condition in neonates with BPD-PH. Characterized by intraluminal narrowing of one or more pulmonary veins, this disorder leads to pulmonary venous hypertension and elevated mPAP [28,41,42,43]. PVS is strongly associated with prematurity and BPD, affecting up to 30% of cases and contributing substantially to morbidity and mortality [41]. Disease onset is often progressive and occurs later in infancy, with severity determined by the number and extent of affected vessels [39,41,42]. Clinical manifestations are frequently nonspecific and may overlap with severe BPD, including persistent hypoxemia, escalating respiratory support, tachypnea, increased work of breathing, and poor growth. Consequently, a high index of suspicion is required for timely diagnosis [44]. Early diagnosis may improve survival, as there are few treatment options when pulmonary veins are completely occluded. Doppler interrogation of pulmonary veins typically demonstrates markedly elevated velocities (>160 cm/s), loss of the normal biphasic or triphasic waveform, and focal turbulent flow across the stenotic segment. Interventional strategies (including catheterization, balloon dilation, or surgical repair) aimed at relieving venous obstruction remain technically challenging with variable outcomes. Until obstruction is addressed, pulmonary vasodilators should be used cautiously, as increased PBF may aggravate pulmonary edema.

Although Type 3a parallels WSPH Group 2 classification, PVS represents a mechanistically distinct entity that is not fully captured by existing classifications. Given their shared pathophysiology of elevated left-sided filling pressures and vulnerability to pulmonary edema from vasodilators, these conditions are collectively categorized as Type 3 BPD-PH within this physiology-based framework.

## 4. Diagnosis of BPD-PH

The clinical signs of moderate to severe BPD and BPD-PH overlap [27]; therefore, it is important to identify BPD-PH with early echocardiography and to allow timely intervention [20,23]. Similar to adults, the WSPH and the EPPVDN recommended definition of PH is based on mPAP of >20 mm Hg at sea level and PVR ≥ 3 Wood units (WU) to identify pre-capillary PH, and PCWP > 15 mmHg for post-capillary PH [23]. While cardiac catheterization remains the diagnostic gold standard, its invasive nature makes these measurements difficult to obtain in preterm neonates. Echocardiography and targeted neonatal echocardiography are the cornerstones of non-invasive diagnosis and longitudinal monitoring of BPD-PH [39].

### 4.1. Echocardiography

**Figure 3:**
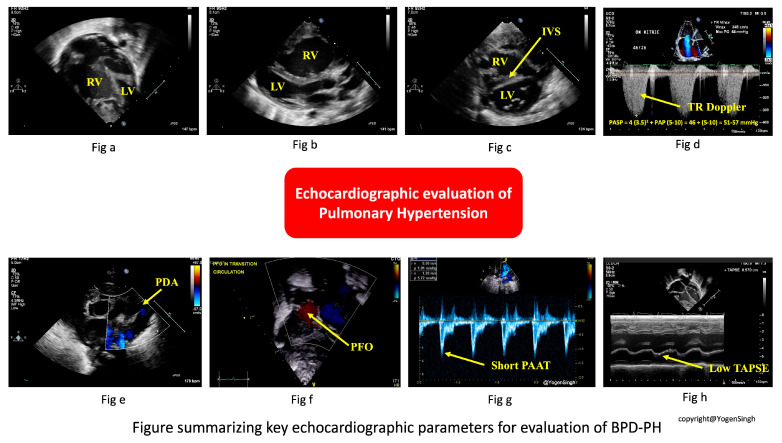
Summary of the common echocardiographic parameters used for evaluation of pulmonary hypertension in infants with BPD-PH. Figures (**a**,**b**) show RV hypertrophy and dilatation in apical 4-chamber view (**a**) and parasternal long axis view (**b**); Figure (**c**) shows IVS flattening in parasternal short axis view and is used for calculating EI; Figure (**d**) shows the estimation of pulmonary artery systolic pressure from peak TR velocity; Figure (**e**) shows right to left shunt across PDA suggesting suprasystemic pulmonary pressure; Figure (**f**) shows bidirectional shunt across PFO; Figure (**g**) shows short PAAT, prolonged RVET, and low PAAT/RVET ratio; and Figure (**h**) shows low TAPSE, suggesting impaired RV systolic function. Abbreviations: EI, eccentricity index; IVS, interventricular septum; PAAT, pulmonary artery acceleration time; PDA, patent ductus arteriosus; PFO, patent foramen ovale; RA, right atrium; RV, right ventricle; RVET, right ventricular ejection time; TAPSE, tricuspid annular plane systolic excursion; TR, tricuspid regurgitation. (Figure courtesy of @Yogen Singh).

The key echocardiographic parameters for evaluating BPD-PH are summarized in Figure 3 and Table 1; serial measurements provide greater insight into disease trajectory and treatment response. Normal reference values in neonates vary with gestational and postnatal age; thus, interpretation that relies on z-scores and trends rather than absolute cut-offs is more beneficial.

(a) Tricuspid Regurgitant (TR) Jet Velocity and RV Systolic Pressure (RVSP)

When present, the TR jet estimates RV systolic pressure (RVSP). Using continuous-wave Doppler aligned through the tricuspid valve in an apical four-chamber (A4C) or RV inflow view, peak TR velocity is measured [38,39]. RVSP is calculated as RSVP = 4 × (TR velocity)^2^ + estimated RA pressure.

In neonates, RA pressure is typically assumed to be 3–5 mmHg. RVSP ≥ 50% of systemic arterial pressure strongly suggests PH. However, TR jet can be absent or incomplete in up to 30–40% of preterm infants and can be inaccurate in the presence of large shunts, necessitating reliance on other indices.

(b) Right ventricular hypertrophy and/or dilatation

RV hypertrophy and/or dilatation may be present, but the findings can be subjective due to the RV geometry. Quantitative measurements are out of the scope of this review.

(c) Interventricular Septal Geometry and LV Eccentricity Index (EI)

Interventricular septal (IVS) flattening is best assessed in parasternal short axis (PASX) imaging at the papillary muscle level. Under normal conditions, the LV appears circular. With RV pressure or volume overload, the septum flattens and produces a “D-shaped” LV. The EI is calculated as the ratio of the LV’s anteroposterior to septo-lateral dimensions in systole and diastole. A systolic EI ≥ 1.30 usually indicates RV pressure overload, while diastolic elevation suggests volume overload.

(d) Pulmonary Artery Acceleration Time (PAAT) and Right Ventricular Ejection Time (RVET) Ratio (PAAT:RVET)

Using pulse-wave Doppler in the RV outflow tract, PAAT is measured as the time interval from onset of systolic flow to peak velocity. RV ejection time (RVET) is measured time from onset to end of systolic flow. Shortened PAAT reflects elevated PVR. The PAAT:RVET ratio corrects for heart rate variability; a ratio < 0.30 indicates increased RV afterload. Pulmonary regurgitation jet velocity can be used to estimate elevated diastolic pulmonary pressure.

(e) Right Ventricular Size and Function

Tricuspid annular plane systolic excursion (TAPSE) is obtained via M-mode through the lateral tricuspid annulus in an A4C view. TAPSE reflects longitudinal RV contractility and systolic function, which decline as afterload rises and ventriculo-arterial coupling fails. Interpretation requires gestational age-adjusted z-scores; a z-score < −2 suggests abnormal RV function. Right ventricular fractional area change (FAC), tissue Doppler, and strain can be excellent tools to assess right ventricular systolic function but detailed description of these methods is beyond the scope of this review article.

(f) Ductal and Atrial Shunt Directionality

Color and spectral Doppler interrogation of PDA (if present) reveals pressure differences between the pulmonary artery and aorta. Bidirectional or right to left shunting in PDA suggests elevated PA. PFO/ASD provides a relative compliance difference between the RV and LV; bidirectional or right-to-left shunting suggest decreased right-sided compliance or increased right-sided pressure. Conversely, large left to right PDA flow can contribute to flow-dependent PH. Hence, flow patterns should cautiously be interpreted in clinical contexts.

(g) Pulmonary Venous Doppler

Sampling the pulmonary veins via suprasternal or high parasternal view with pulse-wave Doppler distinguishes pre-capillary from post-capillary physiology. Normal neonates or those with pre-capillary BPD-PH will have a normal pulmonary vein waveform with forward systolic (S) and diastolic (D) flow with a small atrial reversal (Ar). In post-capillary PH with left-sided heart dysfunction (Type 3a), S is blunted, D is elevated, and Ar is prominent, signaling elevated LA pressure [39]. In post-capillary PH with PVS (Type 3b), there will be increased turbulence flow at the stenotic area. All veins should be interrogated with Doppler tracing, and optimization of the angle of interrogation (i.e., as close to parallel to the flow as possible) is important for interpreting the significance (or lack thereof) of the measured gradients. All four pulmonary veins should be imagined by 2D, color flow, and with Doppler interrogation at the time of every follow-up echocardiogram study for neonates with BPD, as PVS usually evolves over time.

### 4.2. Cardiac Catheterization

Cardiac catheterization remains the gold standard for PH assessment, providing direct hemodynamic measurements, vasoreactivity testing, and collateral vessel detection [19]. Given its invasiveness, management in preterm infants often relies on clinical and echocardiographic findings. Selective use of cardiac catheterization is recommended for therapy escalation, refractory disease, or evaluation of contributory cardiac lesions [19].

### 4.3. Magnetic Resonance Imaging

Cardiac MRI is a non-invasive modality that provides three-dimensional assessment of cardiac structure, function, and blood flow with minimal operator dependence and uniquely allows concurrent evaluation of the lung parenchyma and pulmonary vasculature [48]. However, its use is limited in unstable preterm infants.

### 4.4. Biomarkers

Serum brain natriuretic peptide (BNP) and N-terminal-pro-brain natriuretic peptide (NT-proBNP), which are released by the myocardium in response to wall stress, are the most commonly used biomarkers for monitoring changes in disease status in infants with heart failure or PH. However, these are not specific for RV stress or PH, and only reflect the degree of right heart dilatation [49]. Serial measurements can be easily obtained and are helpful in monitoring disease progression.

## 5. Screening for BPD-PH—Timing and Rationale for Screening

Because the clinical features of BPD and BPD-PH overlap and diseases can be progressive, the optimal timing for screening remains uncertain. However, evidence from multicenter networks and guideline statements (AHA/ATS 2015; ERS/WSPH 2024) supports structured echocardiographic screening at 4 weeks of life (<29 weeks birth gestational age) or 36 weeks postmenstrual age (29–32 weeks birth gestational age), with repeat evaluations every 4–8 weeks if no PH is detected.

**Figure 4:**
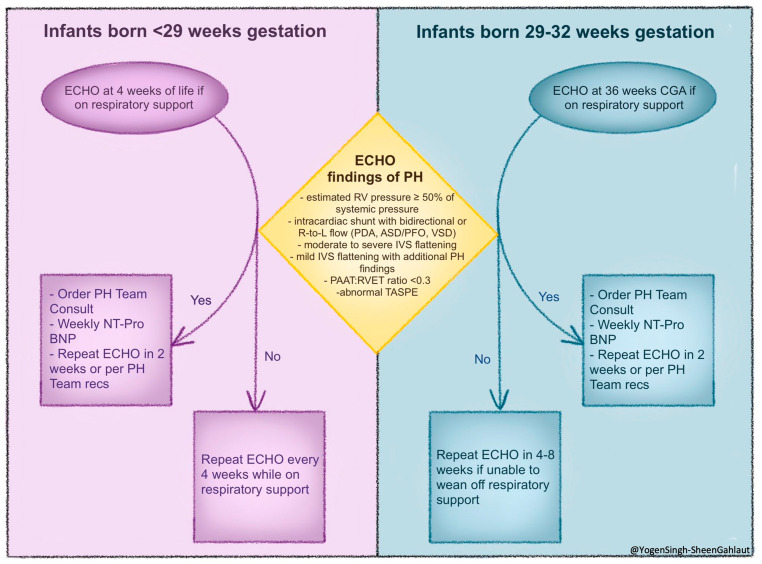
An approach to screening for BPD-PH aimed at early detection of evolving pulmonary vascular diseases in preterm infants with chronic lung disease/BPD. Abbreviations: CGA, corrected gestational age; ECHO, echocardiogram; N-terminal-pro-brain natriuretic peptide (NT-proBNP); IVS, interventricular septum; PAAT:RVET, pulmonary artery acceleration time: right ventricular ejection time ratio; PDA, patent ductus arteriosus; PFO, patent foramen ovale; PH, pulmonary hypertension; RV, right ventricle; RVET, right ventricular ejection time; TAPSE, tricuspid annular plane systolic excursion; VSD, ventricular septal defect. (Figure courtesy of @Yogen Singh-Sheen Gahlaut).

When PH is identified during initial screening, closer surveillance at approximately 2-week intervals and consultation with a PH team are recommended. Infants with severe BPD, prolonged invasive ventilation, or recurrent hypoxemia warrant more frequent monitoring, whereas clinically stable infants on low-flow oxygen can be monitored at standard intervals. Weekly NT-Pro may be a helpful biomarker between echocardiograms. A pre-discharge echocardiogram should be performed in infants requiring ongoing respiratory support, and continued follow-up during early childhood is recommended for those at high risk [20,23,50]. Post-discharge reassessment is advised in the presence of growth faltering, increasing oxygen requirement, recurrent hospitalizations, or clinical deterioration. Referral to a cardiologist or pediatric pulmonary hypertension center should be considered for moderate-to-severe disease, poor treatment response, or diagnostic uncertainty, while cardiac catheterization may be indicated when hemodynamic clarification or escalation of therapy is required. Most centers follow similar risk-based screening strategies, as outlined in Figure 4 below.

## 6. Genetic Determinants, Multi-Omics, and Advances in Diagnosis

As multiple signaling pathways have been implicated in the pathophysiology of BPD-PH, genetic susceptibility and epigenetic programming have been proposed to explain the marked variability in disease severity among neonates born at similar gestational ages and with comparable clinical exposures.

Meta-analysis from multiple studies has demonstrated associations between polymorphisms in the vascular endothelial growth factor (VEGF) signaling pathway for angiogenesis and the risk of BPD [51]. Bhattacharya et al. (2012) linked nitric oxide synthase (NOS) variants to altered vascular tone and increased BPD-PH risk [31]. Epigenetic studies further identified differential DNA methylation of hypoxia-inducible factor (HIF) and oxidative stress response genes in cord blood and tracheal aspirates from neonates who later developed severe BPD [52]. These findings support that antenatal exposures or oxidative stress may genetically alter pulmonary vasculature adaptation [53]. Additional transcriptomic analyses of lung tissue and tracheal aspirates demonstrate the downregulation of angiogenic pathways (VEGF, eNOS) alongside the upregulation of proinflammatory mediators (IL-1β, TNF-α) in BPD-PH compared with BPD alone [27]. The extracellular matrix is essential for lung development. In BPD, disrupted collagen and elastin architecture, supported by proteomic evidence, underscores the role of extracellular matrix dysregulation in disease pathogenesis. [54]. Metabolomic profiling implicates dysregulated sphingolipid and ceramide metabolism, linking lipid signaling to pulmonary vascular remodeling and altered vascular tone [55]. Collectively, these multi-omics signatures help bridge mechanistic insights from bench research to clinically observed phenotypes.

More recently, integrative approaches combining multi-omics data with clinical and imaging markers have been explored to improve risk prediction. Trittmann et al. (2020) demonstrated that a composite of VEGF pathway single-nucleotide polymorphisms and circulating angiogenic protein levels improved the prediction of PH in extremely preterm neonates beyond traditional clinical risk factors [56]. Circulating microRNAs regulating angiogenesis and inflammation are also emerging as promising early biomarkers for BPD-PH [52,57]. In parallel, machine learning models incorporating clinical, echocardiographic, and omics data have shown strong predictive performance for BPD-PH trajectories [58]. Experimental studies further support these observations; in an animal model, Sudhadevi et al. (2021) demonstrated that hyperoxia-induced sphingosine-1-phosphate receptor-1 (S1P1) signaling suppresses ANG-1/TIE-2 and VEGF pathways, linking lipid signaling to impaired angiogenesis and vascular remodeling [33].

In summary, genetic susceptibility, epigenetic programming, and multi-omics signatures provide important insights into the biological heterogeneity of BPD-PH. These biomarkers hold promise for improved risk stratification, the early identification of high-risk infants, and the development of personalized therapeutic strategies that may ultimately transform future diagnostic and management approaches.

## 7. Management of BPD-PH: A Physiology- and Phenotype-Based Approach

Improvements in our understanding of BPD and BPD-PH have transformed our approach from an empiric vasodilator use to a physiology-driven treatment strategy, based on the underlying predominant phenotype as proposed in this review (Type 1–3 BPD-PH). Management of BPD-PH requires a specialized, multidisciplinary team to optimize outcomes in neonates with BPD-PH [59]. Optimizing respiratory care remains the cornerstone of both the prevention and management of BPD-PH.

### 7.1. Optimize Respiratory Support and General Supporting Measures

The Pediatric Pulmonary Hypertension Network (PPHNet), in combination with AHA and ATS, outlines respiratory recommendations for the care of neonates with BPD-PH [20,23]. The emphasis is on identifying and treating the underlying lung pathology as a starting point; this includes optimizing lung mechanics to deliver adequate lung volume, addressing atelectasis and avoiding overexpansion, optimizing non-invasive support if applicable, and reducing lung edema. In addition, assessments should be made of bronchial reactivity, airway clearance and function, and upper airway obstruction. Structural airway abnormalities should be reviewed; these include the identification of tonsillar and adenoidal hypertrophy, subglottic stenosis, tracheobronchial malacia, and vocal cord paralysis. Frequent clinical evaluation, routine blood tests, X-rays, and lung ultrasound will help monitor and optimize these parameters [20,39].

Other important supporting measures include the following:

Oxygen saturation targets—Hypoxemia exacerbates BPD-PH by promoting pulmonary vasoconstriction, thereby increasing PVR. Oxygen saturation (SpO_2_) targets should be maintained between 93 and 97% to prevent hypoxic pulmonary vasoconstriction. Care plans should include review of SpO_2_ histograms from continuous monitoring systems, with a goal of maintaining >80% of daily recordings within the target range. When prolonged or intermittent desaturation is suspected, polysomnography should be considered to characterize hypoxic burden and distinguish obstructive, central, or mixed events [20,23].

Airway evaluation—Airway evaluation is essential in infants with BPD-PH. Bedside flexible bronchoscopy performed by a pulmonologist can differentiate fixed from dynamic large-airway obstruction or collapse, thereby informing ventilator strategies and optimizing airway pressures [20,39].

Subclinical micro-aspiration—Addressing gastroesophageal reflux and subclinical micro-aspiration may further reduce pulmonary inflammation and hypoxemia. Diagnostic evaluation may include upper gastrointestinal contrast studies, impedance or pH monitoring, gastric emptying studies, and formal swallow assessments to identify aspiration risk [20,23].

Comorbidity—Management should also target comorbid conditions that exacerbate pulmonary hypertension physiology, including the treatment of airway malacia or obstruction, prompt management of respiratory infections, evaluation and intervention for pulmonary vein stenosis, and closure of hemodynamically significant cardiac shunts when appropriate [20,23].

Environment—Infants with BPD-PH benefit from a calm, low-stimulation environment with avoidance of excessive physical exertion. Respiratory support should be optimized using chronic care ventilation principles to minimize air hunger and agitation; adequately supported neonates should not require excessive sedation. Finally, emphasis on age-appropriate developmental and family-centered care remains essential to long-term outcomes [60].

They key features of a multidisciplinary approach to the management of BPD-PH have been summarized in Table 2.

**Table 2:** Summary of the multidisciplinary approach to management of BPD-PH. Abbreviations: BPD, bronchopulmonary dysplasia; BPD-PH, bronchopulmonary dysplasia associated pulmonary hypertension; iNO, inhaled nitric oxide.

	Steps in Management	Management Considerations and Action
1.	Establish diagnosis of BPD-PH	Echocardiography remains the best choice for screening and establishing diagnosis of BPD-PH and determining its phenotype. Cardiac catheterization is the gold standard for diagnosis of PH, determining its phenotype and establishing if there is iNO pulmonary vasodilator responsiveness.
2.	Multidisciplinary team	Collaborative approach among multidisciplinary team including neonatologist, pediatric cardiologist, pediatric pulmonary hypertension team, pulmonologists, respiratory therapist, pharmacist, gastroenterologist, nursing staff, dieticians, occupational therapists, physical therapists, and ear/nose/throat (ENT) team, and mostly importantly family engagement.
3.	Other key diagnostics	Lung ventilation and oxygenation monitoring, including serial blood gases for monitoring hypocapnia and hypercapnia; continuous pulse oximetry for oxygenation; chest X-ray to evaluate lung expansion. Echocardiography—serial evaluations to monitor PH disease progression and response to therapy. Upper gastrointestinal series, impedance, pH monitoring, and gastric emptying time, or swallow study—to rule of gastro-esophageal reflux. Sleep study—to evaluate for any obstructive, central or mixed process causing extended periods of hypoxia.
4.	Key therapeutic therapies	Optimizing respiratory support and lung protective ventilation strategies. Maintaining optimal oxygen saturation—pulse oximetry to maintain target oxygen saturation levels (93–97%). Pulmonary vasodilators and other cardioactive medications (see text for details on types of pulmonary vasodilators)—a summary of phenotype-based management is described in Table 3. Diuretics for pulmonary edema. Optimizing nutrition, somatic growth, and neurodevelopmental intervention. Specific interventions: Closure of underlying cause of left to right shunt, stenting for pulmonary vein, treatment for gastro-esophageal reflux, tracheostomy and/or gastrostomy, and home oxygen.

**Table 3:** Summary of pulmonary vasodilators. Abbreviation: NO nitric oxide; cAMP, cyclic adenosine monophosphate; cGMP, cyclic guanosine monophosphate; PDE3, phosphodiesterase type 3; PDE5, phosphodiesterase type 5; PH pulmonary hypertension.

Agent	Pathway	Primary Role	Key Cautions
Oxygen	Increased Hypoxic vasoconstriction	Foundational therapy	Avoid hyperoxia
Inhaled Nitric Oxide	NO–cGMP	Acute pulmonary vasodilation	Variable response; short-term use
Sildenafil	PDE5 inhibitor → Increased cGMP	Common chronic therapy; supports iNO weaning	Risk of systemic hypotension
Milrinone	PDE3 inhibitor → Increased cAMP	Improves cardiac output with ventricular dysfunction	Hypotension; may worsen pulmonary edema
Bosentan	Endothelin receptor antagonist	Chronic therapy in selected cases	Hepatotoxicity
Iloprost, Treprostinil, Epoprostenol	Prostacyclin analogs—increased cAMP	Severe or refractory PH	Limited neonatal data; complex delivery

### 7.2. Pulmonary Vasodilators

Oxygen therapy is the first-line pulmonary vasodilator in BPD-PH and should be carefully titrated to avoid oxidative injury from hyperoxia [20,44]. There are three core vasoregulatory pathways used for targeted PH treatment when clinically indicated—NO-sGC, endothelin-1 (ET-1), and prostacyclin-IP-cAMP pathway (Table 3).

- **NO-sGC-cGMP pathway:**
  1. *Inhaled nitric oxide (iNO)* is the first-line therapy which is a rapid-acting, selective pulmonary vasodilator, which is delivered directly to the pulmonary microvasculature. It diffuses into the smooth muscle endothelium and stimulates soluble guanylate cyclase (sGC), increases intracellular cyclic guanosine monophosphate (cGMP), and relaxes vascular smooth muscle. iNO is briefly used to manage an acute PH crisis by temporarily improving V/Q mismatch due to its variable pulmonary vasodilator response in this cohort [20,61].
  2. *Sildenafil* is a phosphodiesterase type 5 (PDE5) inhibitor and is one of the most commonly used therapies in neonates with BPD-PH. By preventing cGMP degradation, it enhances nitric oxid-mediated vasodilation. Sildenafil may be used as primary therapy, as an adjunct to iNO, or to facilitate weaning from iNO. Additionally, it may reduce pulmonary vasoreactivity and attenuate adverse vascular remodeling [61].
  3. *Milrinone* is a PDE3 inhibitor and it increases cyclic adenosine monophosphate cAMP. Milrinone increases cardiac contractility, improves ventricular function, and reduces systemic resistance and PVR. It only has an intravenous formulation, and it may cause hypotension and worsening pulmonary edema.
- **Endothelin-1 Pathway (ET-1):**
  1. *Bosentan* is an ET-1 receptor antagonists which is a common chronic therapy for PH ET-1 that promotes smooth muscle cell proliferation and vasoconstriction by acting on two receptors, ETa and ETb. ETb additionally mediates vasodilation by the release of NO and PGI2 from endothelial cells. It is associated with hepatotoxicity and needs close monitoring of liver function tests. Other less hepatotoxic ET-1 receptor antagonists agents have been less studied in infants [19,20].
- **Prostacyclin-IP-cCAMP pathway:**
  1. *Iloprost*, *treprostinil*, *and epoprostenol* are arachidonic acid metabolites, prostaglandins, and prostacyclin analogs which cause vasodilation by activating adenylate cyclase, increasing intracellular cAMP, opening Calcium2+ channel-activated potassium channels, and relaxing smooth muscle [61]. There is limited evidence to prescribe prostacyclin analogs as a therapy for BPD-PH infants [20,61].

### 7.3. Phenotype-Specific Strategies

Management beyond lung optimization depends on the dominant PH phenotype, as outlined in the previous section (Table 4):

## 8. Future Directions

Advances in developmental biology and molecular signaling are shifting the focus of BPD-associated pulmonary hypertension toward precise, mechanism-based therapies. Improved understanding of pulmonary vascular–alveolar interactions may enable strategies that promote normal lung growth rather than relying solely on vasodilator therapy. Emerging approaches—including single-cell transcriptomics, regenerative therapies, and stem cell-derived or exosome-based treatments—show promise but remain investigational [62,63,64,65,66].

However, major gaps persist, including limited randomized data, uncertainty regarding optimal timing of intervention, and a lack of validated predictors of treatment response. Future priorities include multicenter trials, standardized phenotyping, integration of advanced imaging and biomarkers, and longitudinal studies to better define outcomes.

## 9. Conclusions

BPD and BPD-PH remain significant causes of morbidity and mortality in extremely preterm infants. This review supports a physiology-based approach that emphasizes early phenotypic recognition and discusses echocardiographic findings and targeted intervention. Advancing care will require collaborative research efforts to refine diagnostic strategies, evaluate emerging therapies, and improve long-term cardiopulmonary outcomes.

## Figures and Tables

**Table 1:** Summary of the common echocardiographic parameters and thresholds for determining BPD-PH and its severity [21,26,44,45,46,47]. Abbreviations: CW, continuous wave; EI, eccentricity index; IVS, interventricular septum; LA, left atrium; LV, left ventricle; PAAT, pulmonary artery acceleration time; PDA, patent ductus arteriosus; PFO, patent foramen ovale; PR, pulmonary valve regurgitation; PSAX, parasternal short axis view; PVR, pulmonary vascular resistance; PW, pulse wave; RA, right atrium; RAP, right atrial pressure; RV, right ventricle; RVET, right ventricular ejection time; RVOT, right ventricular outflow track; RVSP, right ventricular systolic pressure; TAPSE, tricuspid annular plane systolic excursion; TR, tricuspid regurgitation.

Echo Parameter	Measurement	Physiology	Neonatal Target	Suggests PH or Its Severity
TR jet velocity	CW Doppler to measure peak TR velocity; RVSP ≈ 4 × (V^2^) + RAP	RV systolic pressure surrogate	<2.8 m/s (can be absent)	>2.8−3.0 m/s in presence of other parameters and clinical context
EI	PSAX to assess LV shape	RV pressure or volume overload	Round LV	Variable degree of septal flattening and D-shaped LV
PAAT	PW Doppler on RVOT to measure time to peak velocity	↑ PVR reduces PAAT	>70 ms (by 3 months)	Mild 60−70 ms; Moderate 45–60 ms; Severe < 45 ms
TAPSE	M-mode lateral tricuspid annulus	RV systolic function from longitudinal shortening	>8 mm	<8 mm; Severe < 6 mm (for term infants)
RV/LV end-diastolic ratio	PSAX	RV dilation from load	<0.6	Mild 0.6−0.7; Moderate: 0.7−0.9; Severe: ≥0.9 severe
RA and RV size	Qualitative/area index	Chronic pressure load	Normal	Enlarged RA
Pulmonary regurgitation gradient	CW Doppler if PR present	Mean PA pressure surrogate	Low/undetectable	Determine severity of mean or end-diastole PAP
Pulmonary vein Doppler	PW Doppler of all 4 veins	Increased velocity with left sided dysfunction or turbulence flow at stenotic vein	Normal flow pattern and low laminar velocity	Identify pulmonary vein stenosis

**Table 4:** Summary of phenotype-based BPD-PH recognition and targeted management [38]. ASD, atrial septal defect; BPD, bronchopulmonary dysplasia; BPD-PH, bronchopulmonary dysplasia associated pulmonary hypertension; ECHO, echocardiogram; LA, left atrium; LV, left ventricle; VSD, ventricular septal defect; PDA, patent ductus arteriosus; PFO, patent foramen ovale; PBF, pulmonary blood flow; PCWP, pulmonary capillary wedge pressure; PH, pulmonary hypertension.

	Type 1 Increased PVR	Type 2 Increased PBF	Type 3 Increased PCWP
Diagnostic clues to BPD-PH phenotype			
Key features	This phenotype vascular tone-driven with relatively preserved vascular architecture. Improvement occurs after oxygenation optimization as oxygen is a potent vasodilator, and gentle ventilation is common. If echocardiographic indices and NT-proBNP remain abnormal despite optimized care, selective pulmonary vasodilators may be introduced (e.g., sildenafil) [20,21,23].	Timely closure of hemodynamically significant left-to-right shunts is critical to reduce pulmonary overcirculation before irreversible pulmonary vascular remodeling occurs. Diuretic therapy may help alleviate pulmonary edema. The use of iNO or other vasodilators may worsen the condition by further increasing pulmonary blood flow and should be avoided. These neonates often require careful evaluation with cardiac catheterization to provide a detailed hemodynamic assessment and guide targeted intervention.	Management focuses on treating LV dysfunction while avoiding pulmonary vasodilators that may exacerbate pulmonary edema. Pulmonary vasodilators increase BPF and may worsen pulmonary edema and venous congestion. Milrinone may be beneficial in the setting of LV dysfunction (Type 3a); however, it should be used cautiously when PVS is present, as it may worsen pulmonary venous congestion. Once PVS is identified, a thorough evaluation is required to guide targeted interventions, including catheter-based therapies such as stenting [20,39].
Classic history	History of BPD with persistent respiratory support, often worsening of oxygen requirement, work of breathing and respiratory support.	History of persistent PDA, PFO/ASD, VSD, or aorto-pulmonary collaterals, any other left-to-right shunt.	3a—Worsening of LV dysfunction or mitral valve pathology 3b—History of worsening respiratory support, increased oxygen requirement and work of breathing, especially in infants with small for gestation age and BPD.
Chest X-ray	Non-homogenous changes on chest X-ray consistent with BPD.	Non-homogenous changes with worsening of pulmonary edema on chest X-ray.	Worsening of pulmonary edema on chest X-ray in the setting of non-homogenous changes from BPD.
Echo findings	Classical signs of PH on ECHO.	Diagnosis of left to right shunt with worsening of heart dilatation and other ECHO parameters of PH.	3a—Worsening of LV dysfunction, presence of mitral regurgitation and LA enlargement 3b—Evidence of pulmonary vein stenosis and other ECHO parameters of PH.
Guide to targeted specific therapy			
Targeted specific therapy	Optimize ventilation, pulmonary vasodilators; optimize hemodynamic support.	Optimize ventilation, diuretics, and treatment of specific lesion; optimize hemodynamic support; avoid vasodilators.	Optimize ventilation, treatment of specific lesions such as pulmonary vein stenosis or improve LV function with lusitropic drugs such as milrinone; avoid vasodilators.

## Data Availability

Data sharing is not applicable.
